# Protective Effect of Peony (*Paeonia ostii)* Flower Extract Against Tape Stripping-Induced Skin Barrier Impairment in Mice

**DOI:** 10.3390/molecules31010062

**Published:** 2025-12-24

**Authors:** Ruiying Yang, Jicheng Yang, Gaiying He, Yusheng Zhang, Xue Jiang, Jiyong Wang, Hongjun Yang, Chengxiang Shang

**Affiliations:** 1 Beijing Key Laboratory of Traditional Chinese Medicine Basic Research on Prevention and Treatment for Major Diseases, Experimental Research Center, China Academy of Chinese Medical Sciences, Beijing 100700, China; 15651901275@163.com (R.Y.); yjc18143609206@163.com (J.Y.); gaiyinghe2012@163.com (G.H.); yushengzhang271727@foxmail.com (Y.Z.); 2112218003@stmail.ujs.edu.cn (X.J.); 2 China Traditional Chinese Medicine Co., Ltd., Beijing 100089, China

**Keywords:** peony flower extract, skin barrier, proteomics, cornified envelope, tight junctions

## Abstract

**Background:** Skin barrier dysfunction leads to increased transepidermal water loss (TEWL), inflammation, and compromised skin protection. While *Paeonia ostii* (peony) flowers are recognized in traditional Chinese medicine for their reducing melanin synthesis, anti-inflammatory, and anti-aging effects, their role in repairing skin barrier damage has not been fully explored. **Methods:** We investigated the therapeutic potential of peony flower extract (PFE) in the tape-stripping-induced mouse model with skin barrier damage. Skin surface imaging, hydration measurements, H&E, proteomics, qPCR, and immunofluorescence were applied to clarify the potential mechanism of PFE in attenuating skin barrier impairment. **Results:** PFE significantly reduced erythema, TEWL, and edema while restoring epidermal architecture. Proteomics analysis identified cornified envelope formation and tight junction assembly as essential mechanisms in skin barrier repair. It increased the expression of key skin barrier proteins, including filaggrin (Flg), involucrin (Ivl), loricrin (Lor), claudin-1 (Cldn1), tight junction protein 1 (Tjp1), and occludin (Ocln). **Conclusions:** This study demonstrates that PFE restores skin barrier integrity by upregulating key structural proteins within the cornified envelope and tight junction. These findings suggest that PFE is a promising therapeutic candidate for skin barrier repair, with high potential in translational medicine applications.

## 1. Introduction

The skin constitutes the body’s primary defense against external insults—including pathogens, environmental aggressors, and ultraviolet radiation—while concurrently preventing transepidermal water and solute loss [1]. This critical protective function is mediated by a multi-layered barrier system encompassing the stratum corneum, tight junctions, and microbial, chemical, and immune barriers [2,3]. Dysfunction in any component of this system may trigger xerosis, inflammatory responses, and systemic pathological manifestations [4,5]. Specifically, barrier impairment elevates skin permeability, increases transepidermal water loss (TEWL), reduces hydration levels, elevates pH, and culminates in clinical sequelae including erythema, desquamation, fissuring, pruritus, and inflammation [6]. Notably, dermatoses such as psoriasis, atopic dermatitis (AD), acne, and melasma are intrinsically linked to barrier dysfunction, underscoring the critical need to preserve barrier integrity [7].

Research on therapies for damaged skin barrier repair has intensified across academic and industrial sectors, driven by the need to develop effective strategies. However, the requirement for gentle, non-toxic interventions poses a significant therapeutic challenge in dermatological practice. Traditional Chinese medicine (TCM), characterized by its favorable safety profile and low toxicity, offers a promising alternative. Consequently, TCM compounds have been explored as multi-targeting agents for skin barrier impairment, exemplified by *Centella asiatica* (L.) Urban [8], *Aloe barbadensis* Miller [9], and *Panax ginseng* C. A. Mey [10]. Shedoeva et al. [11] systematically evaluated the wound-healing potential of 36 botanical species, identifying peony as a high-value candidate. Supporting this, an in vitro study demonstrated that peony extract dose-dependently enhances viability in both HaCaT keratinocytes and primary human dermal fibroblasts [12]. Despite this therapeutic potential, current dermatological research on peony focuses predominantly on anti-aging and whitening effects, while its mechanistic role in skin barrier repair remains underexplored.

*Paeonia ostii*, one of the several wild species of Chinese tree peony, is a woody shrub that is widely distributed in China [13]. Since 2013, *Paeonia ostii* flowers have been officially designated as a new resource food by China’s Ministry of Health [14]. Toxicological studies confirm an absence of genotoxicity, supporting its safety profile [15]. While numerous studies report reducing melanin synthesis, antioxidant, anti-inflammatory, and antibacterial activities of peony flowers, the specific role of *P. ostii* in repairing skin barrier damage remains unelucidated. Therefore, this work was aimed at investigating the effect of the extract from *Paeonia ostii* peony flower on barrier repair of the damage induced by tape-stripping, along with the exploration of the putative underlying mechanisms of repair.

## 2. Results

### 2.1. Preparation and HPLC Analysis of PFE

**Figure 1:**
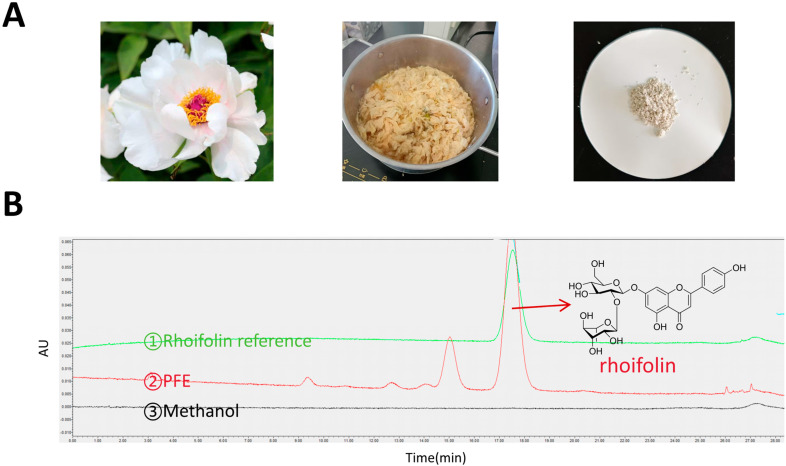
Preparation and HPLC Analysis of PFE. (**A**) The representative image of fresh peony flowers, dried peony flowers, and powder of extract PFE. (**B**) The HPLC chromatogram of the samples.

*Paeonia ostii* peony flowers feature a single-petal structure with 10–15 small white petals arranged in two to three whorls, forming a flat to semi-flat flower that exposes the central stamens and pistils. The dried flowers, sourced from Heze, China, were traditionally sun-dried to a uniform yellow-brown color. The optimal extraction conditions determined were: a solid-to-liquid ratio of 1:20, decoction for 4 h, followed by cooling at 4 °C for 24 h at pH 6–7. The resulting solid was air-dried and ground into a light yellow powder, designated peony flower extract (PFE) (Figure 1A).

The flavonoid content in peony flowers is significantly higher than that in most ornamental flower varieties, as well as in other parts of the plant, such as root bark and branches. The compositional characteristics of flavonoids have thus become critical markers for the identification and quality assessment of peony varieties [16,17]. HPLC analysis confirmed rhoifolin as the major constituent, with a relative peak area of 75.48% ± 0.36% (n = 5). Based on this result, a relative peak area of rhoifolin ≥ 75% was established as a criterion for qualifying the quality of the extract (Figure 1B).

The HPLC analysis was performed under the following conditions: a C18 reversed-phase column (Agilent Technologies Inc., Santa Clara, CA, USA); mobile phase consisting of methanol (A) and 0.1% formic acid aqueous solution (B), with an isocratic elution program maintaining 35% A from 0 to 30 min; flow rate of 1.0 mL/min; column temperature set at 25 °C; detection wavelength of 338 nm (determined by full-wavelength UV scanning); and an injection volume of 10 μL.

### 2.2. PFE Facilitated the Restoration of the Dorsal Skin Appearance

**Figure 2:**
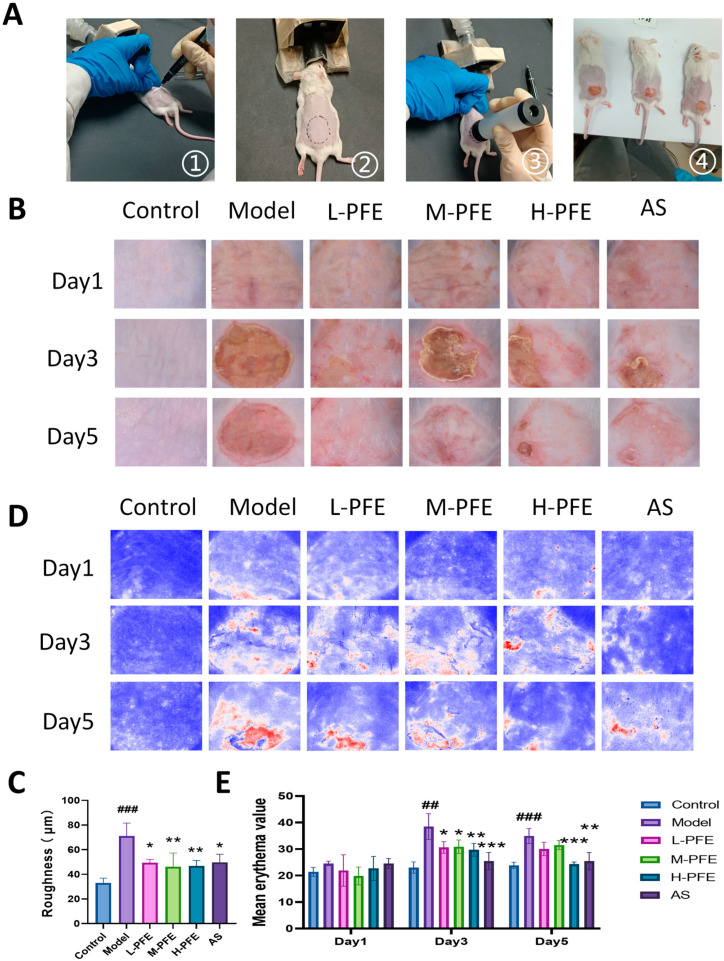
PFE facilitated the restoration of the dorsal skin appearance: (**A**) The modeling process of tape stripping. (**B**) Representative images showing mice’s dorsal skin surfaces. (**C**) Statistical analysis of skin roughness on Day 5. (**D**) Representative images showing erythema on the dorsal surface of mice. (**E**) Statistical analysis of skin erythema. Values are expressed as mean ± SD. One-way ANOVA with Tukey’s analysis was used. # Indicates a significant difference compared with the control group (### *p* < 0.001 and ## *p* < 0.01). * Indicates a significant difference compared with the model group (*** *p* < 0.001, ** *p* < 0.01, and * *p* < 0.05).

The tape stripping method represents a classical, well-established approach for inducing skin barrier impairment in dermatological research (Figure 2A). Based on preliminary experiments and literature validation, a TEWL value of 200 g·m^−2^·h^−1^ indicated effective skin barrier impairment, confirming model readiness for subsequent experiments [18,19]. Model group mice exhibited characteristic skin barrier impairment on Day 1, presenting erythema and dryness. By Day 3, these manifestations progressed to exacerbated erythema, edema, and scab formation. Persistent erythema and edema persisted through Day 5 despite partial scab resolution. In contrast, PFE treatment showed dose-dependent phenotypes, with medium dose (M-PFE) and high dose (H-PFE) groups demonstrating pronounced therapeutic efficacy (Figure 2B,C). Quantification of skin arithmetic average roughness and mean erythema value in H-PFE treatment was significantly increased compared to M-PFE treatment, which indicates more severe damage for both metrics. The results confirmed PFE’s significant dose-dependent efficacy in accelerating skin barrier restoration, reducing erythema severity, and improving skin smoothness. The PFE exhibited the most robust therapeutic effects (Figure 2D,E).

### 2.3. PFE Reduced TEWL and Increased SH of the Dorsal Skin

TEWL, a well-established parameter for skin barrier impairment, measures non-visible water evaporation through the stratum corneum. Higher TEWL indicates poorer barrier function. Surface hydration (SH) reflects skin moisturizing capacity, with lower SH values signifying reduced moisturizing ability [18,19].

**Figure 3:**
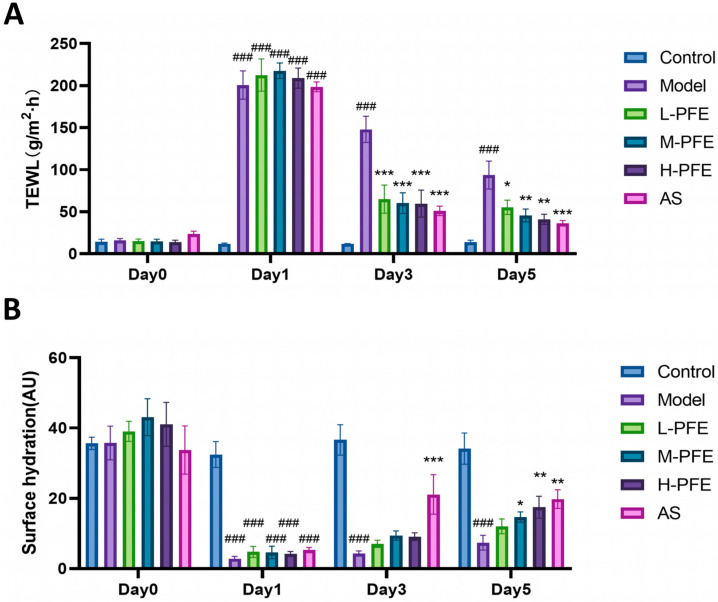
PFE reduced TEWL and increased SH of the dorsal skin: (**A**) Statistical analysis of TEWL measurements in the skin of mice across experimental groups at Baseline (Day 0) and on Days 1, 3, and 5 post-modeling. (**B**) Statistical analysis of SH measurements in the skin of mice across experimental groups at Baseline (Day 0) and on Days 1, 3, and 5 post-modeling. Values are expressed as mean ± SD. One-way ANOVA with Tukey’s analysis was used. # Indicates a significant difference compared with the control group (### *p* < 0.001). * Indicates a significant difference compared with the model group (*** *p* < 0.001, ** *p* < 0.01, and * *p* < 0.05).

On Day 0, no significant intergroup differences in TEWL or SH were observed. By day 1 post-modeling, experimental groups exhibited significantly increased TEWL and decreased SH compared to controls, indicating enhanced water loss. On Day 3, the model group maintained significantly higher TEWL and lower SH than controls. Notably, the AS group and all PFE1 dose groups demonstrated significantly lower TEWL than the model group. On Day 5, all PFE dose groups showed significantly lower TEWL and higher SH than the model group (Figure 3A,B), with efficacy comparable to AS. These results suggest that PFE facilitates skin barrier repair by reducing TEWL and enhancing SH.

### 2.4. PFE Facilitated the Repair of Edematous Conditions and the Epidermal Tissue Structure

**Figure 4:**
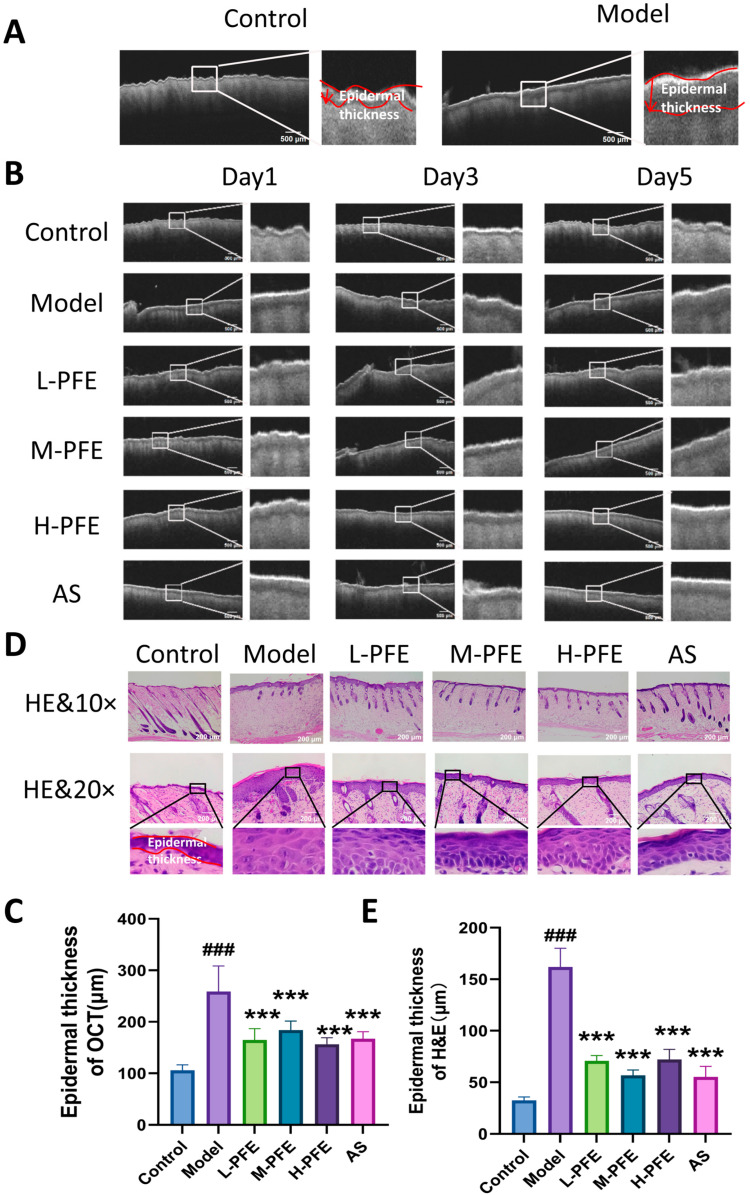
PFE facilitated the repair of edematous conditions and the epidermal tissue structure: (**A**) Representative OCT images of the normal skin and model skin. Epidermal thickness is highlighted by the red line. (**B**) Representative OCT images of mouse skin tissue on Days 1, 3, and 5 post-modeling. (**C**) Statistical analysis of skin thickness based on OCT imaging. (**D**) Representative H&E-stained longitudinal sections of skin on Day 5 post-modeling. Epidermal thickness is highlighted by the red line. (**E**) Statistical analysis of epidermal thickness based on H&E Imaging. Values are expressed as mean ± SD. One-way ANOVA with Tukey’s analysis was used. # Indicates a significant difference compared with the control group (### *p* < 0.001). * Indicates a significant difference compared with the model group (*** *p* < 0.001).

OCT monitoring of skin repair revealed distinct structural differences between the experimental group. Control skin exhibited a well-defined dermal–epidermal junction (DEJ) with tight interlayer adhesion. In contrast, the model group displayed significant epidermal thickening and a signal void at the DEJ, indicative of edema fluid accumulation, altering light scattering. Epidermal thickness quantification was used to assess edema severity (Figure 4A).

On Day 1, epidermal thickness of OCT (reflecting edema severity) was significantly greater in the model group compared to the control group. By Day 3, epidermal thickness remained elevated in the model group versus controls. However, PFE treatment significantly reduced edema compared to the untreated model group. On Day 5, epidermal thickness in the model group persisted above control levels, while PFE treatment further reduced edema, demonstrating efficacy comparable to the positive control drug AS (Figure 4B,C).

Histological assessment of skin structure in the tape stripping model, performed via HE staining, revealed that tape stripping induced pathological hyperplasia of the stratum corneum, with evident hyperkeratosis. Additionally, a notable increase was observed in both the number and volume of spinous layer cells. Conversely, in the PFE intervention group, the number of spinous cells decreased, and their morphology gradually normalized (Figure 4D). Epidermal thickness was also significantly reduced in the PFE group, with a repair effect comparable to that of the positive control AS group (Figure 4E).

### 2.5. Proteomic Changes in Skin Following the PFE Intervention

**Figure 5:**
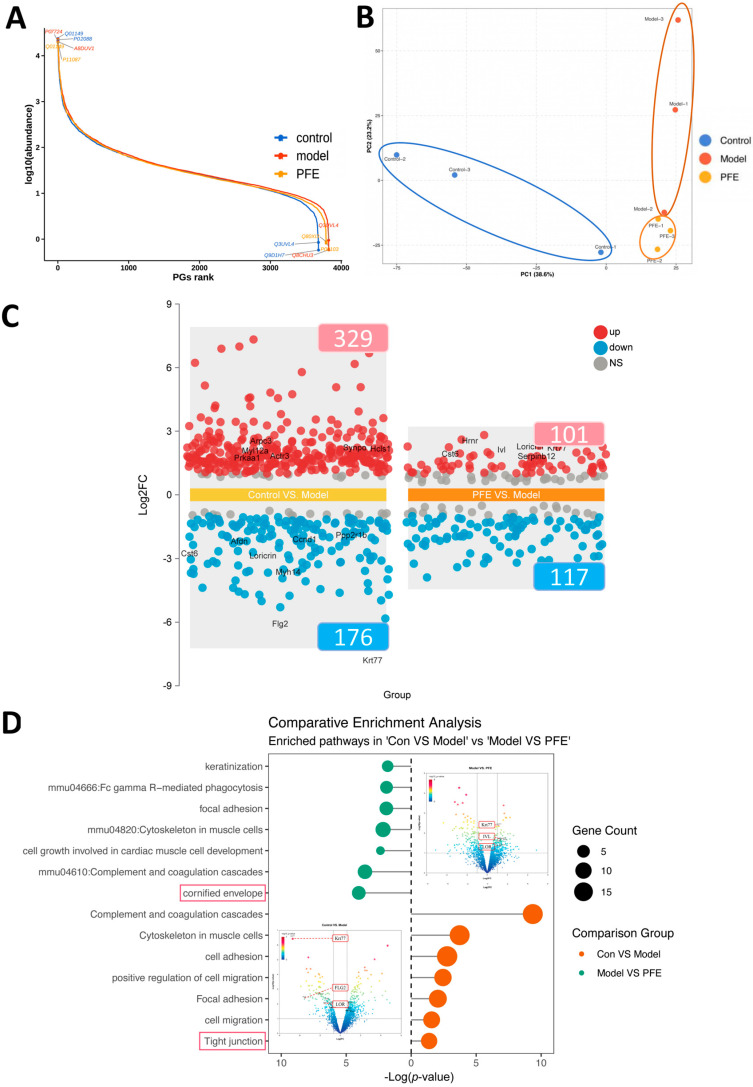
Proteomic changes in skin following the PFE intervention: (**A**) PGs rank graph. (**B**) PCA of regulatory proteins. (**C**) Volcanic map of up- and downregulated proteins. (**D**) Pathway enrichment of differentially expressed proteins.

Proteomics analysis was performed to investigate the molecular mechanisms. The PGs rank graph shows that the data of the control, model, and PFE groups all span a wide range and have smooth, continuous curves, indicating that the mass spectrometry data distribution is stable and has a wide dynamic range, which can be used for subsequent analysis (Figure 5A). Principal Component Analysis (PCA) showed that there was a significant difference between the model group and the control group, and this change was significantly improved in the PFE group (Figure 5B). A total of 4106 proteins were identified. Among the differentially expressed proteins (DEPs; fold change >1.5 or <0.7, *p* < 0.05), the model group exhibited 329 upregulated and 176 downregulated proteins compared to the normal group. In contrast, relative to the model group, the PFE group showed 101 upregulated and 117 downregulated proteins (Figure 5C). Notably, tight junctions (TJs) and cornified envelope (CE) related proteins (including filaggrin2, loricrin, and involucrin) were significantly enriched among these DEPs, consistent with our prior protein-level findings (Figure 5D).

### 2.6. PFE Enhanced Both mRNA and Protein Expression of Flg, Ivl, and Lor in the Tape Stripping Mouse Model

**Figure 6:**
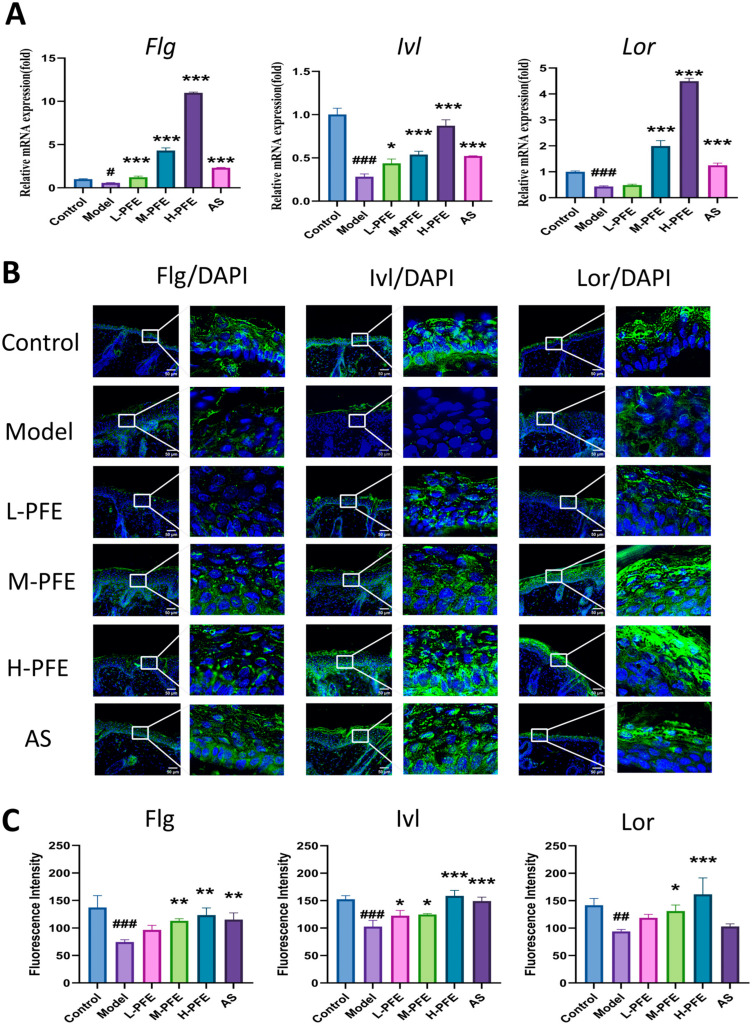
PFE enhanced both mRNA and protein expression of Flg, Ivl, and Lor in the tape stripping mouse model: (**A**) The *Flg*, *Ivl*, and *Lor* mRNA levels in skin tissues were assayed after 5 days of treatment by qPCR; *Gapdh* mRNA expression results served as a loading control. (**B**) Representative IF images depicting Flg, Ivl, and Lor protein expression in skin on Day 5 post-modeling. (**C**) Quantitative analysis of fluorescence intensity based on IF. Values are expressed as mean ± SD. One-way ANOVA with Tukey’s analysis was used. # Indicates a significant difference compared with the control group (### *p* < 0.001, ## *p* < 0.01, and # *p* < 0.05). * Indicates a significant difference compared with the model group (*** *p* < 0.001, ** *p* < 0.01, and * *p* < 0.05).

As core structural components of the CE, Flg, Ivl, and Lor confer mechanical resilience through transglutaminase-mediated cross-linking with keratins K1, K10, and desmosomal proteins, playing crucial roles in skin barrier integrity [20,21]. Quantitative analysis revealed a significant decrease in *Flg*, *Ivl*, and *Lor* mRNA expression in the model group versus controls. PFE intervention restored expression to varying degrees, with high-dose PFE (H-PFE) demonstrating the most pronounced effect—increasing gene expression levels 2- to 10-fold relative to the model group. Notably, H-PFE induced significantly increased *Flg*, *Ivl*, and *Lor* mRNA than the positive control (AS) group (Figure 6A).

Flg, Ivl, and Lor proteins are mainly localized to the epidermal granular layer. Compared with the control group, all the protein expression of Flg, Ivl, and Lor was significantly decreased in the model group. However, the treatment of H-PFE significantly increased the signal of all proteins. Specifically, H-PFE elicited superior upregulation of Lor protein compared to the AS group, while exerting comparable upregulatory effects on Flg and Ivl expression relative to AS (Figure 6B,C). The immunofluorescence (IF) signal of the protein signal level was consistent with the gene expression level.

### 2.7. PFE Enhanced Both mRNA and Protein Expression of Cldn1, Tjp1, and Ivl in the Tape Stripping Mouse Model

**Figure 7:**
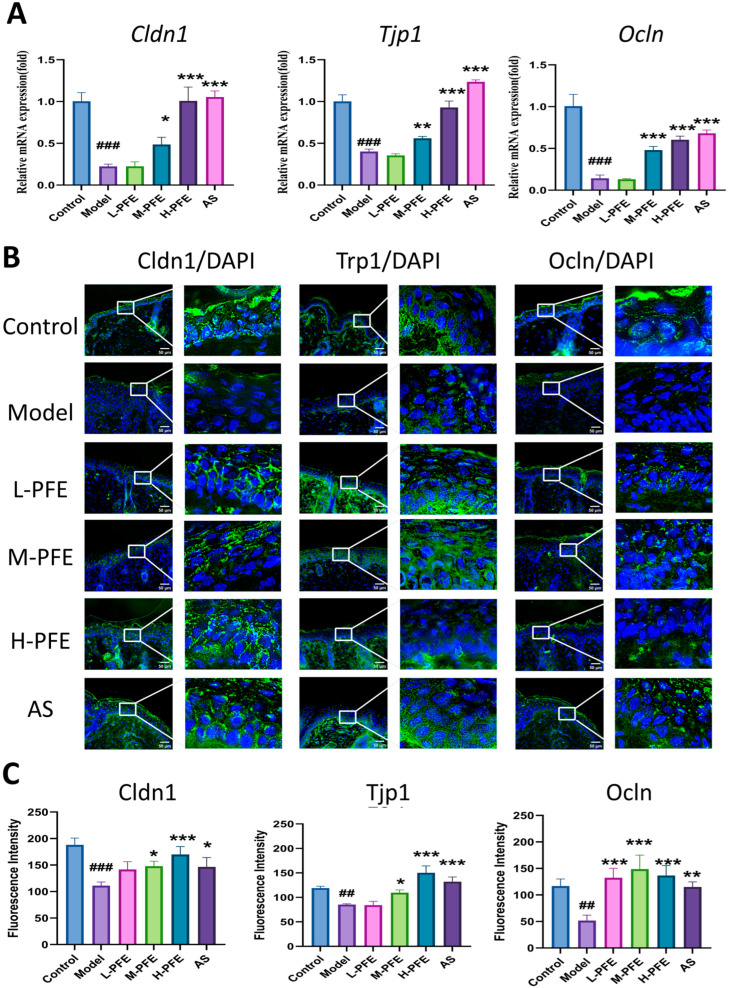
PFE enhanced both mRNA and protein expression of Cldn1, Tjp1, and Ivl in the tape stripping mouse model. (**A**) The *Cldn1*, *Tjp1*, and *Ocln* mRNA levels in skin tissues were assayed after 5 days of treatment by qPCR; *Gapdh* mRNA expression results served as a loading control. (**B**) Representative IF images depicting Cldn1, Tjp1, and Ocln protein expression in skin on Day 5 post-modeling. (**C**) Quantitative analysis of fluorescence intensity based on IF. Values are expressed as mean ± SD. One-way ANOVA with Tukey’s analysis was used. # Indicates a significant difference compared with the control group (### *p* < 0.001 and ## *p* < 0.01). * Indicates a significant difference compared with the model group (*** *p* < 0.001, ** *p* < 0.01, and * *p* < 0.05).

Epidermal tight junction complexes, composed of transmembrane proteins (including Cldn1-7, (occludin) Ocln, and Tjp1/Tjp-2/Tjp-3), activate cellular processes essential for skin barrier integrity [22]. The model group exhibited significantly reduced mRNA expression of *Cldn1*, *Tjp1*, and *Ocln*. PFE intervention dose-dependently reversed this downregulation, with the high-dose PFE group elevating mRNA levels 2- to 5-fold relative to the model group (Figure 7A).

At the protein level, Cldn1 (localized to keratinocyte tight junctions) was markedly lower in the model group versus controls. Both medium- and high-dose PFE (M/H-PFE) groups significantly increased Cldn1 protein expression compared to the model group, exceeding the effect of the positive control AS group. Tjp1 and Ocln formed characteristic membrane networks in the granular layer. Their expression was significantly decreased in the model group, while H-PFE intervention effectively restored both proteins to near-control levels (Figure 7B,C).

## 3. Discussion

Skin barrier impairment constitutes the pathological foundation of numerous chronic dermatoses, necessitating therapeutic interventions with low-irritancy agents. *P. ostii* flower, a traditional Chinese medicine with significant therapeutic potential, demonstrates a favorable safety profile in dermatological applications. Our study is the first to demonstrate that PFE effectively ameliorates tape-stripping-induced skin barrier disruption. Proteomic analysis identified significantly differentially expressed proteins associated with this repair process. Biological experiments and proteomic data collectively indicate that the pathways involving these proteins critically regulate key structural components of the CE and TJs.

The integrity of the epidermal barrier is vital for protection from pathogens and the reduction in TEWL [23]. Studies have shown that impaired skin barrier function is a hallmark feature of various skin diseases [24]. Abnormal skin barrier properties have been reported in several dermatological disorders, including atopic dermatitis [25], psoriasis [26], contact dermatitis [27], and acne [28]. PFE, a traditional Chinese medicine, has a long history of application. Research has indicated its dermatological effects in reducing melanin synthesis and exerting antioxidant activity [29,30,31]. However, there have been few reports on its role in skin barrier repair. This study provided the first evidence that PFE significantly upregulated the expression of key skin barrier components CE and TJs induced in mice with skin barrier damage. This upregulation consequently reduced transepidermal water loss and promoted the restoration of skin barrier function.

Tape stripping represents a well-established experimental approach for inducing skin barrier disruption in animal studies [32,33,34], in which TEWL serves as an essential evaluation method for monitoring functional restoration of the skin barrier. In this study, a skin barrier injury model was established by repeatedly stripping tape from the dorsal skin of mice, and TEWL measurements were used as a primary objective indicator throughout the experimental process. An intact skin barrier is characterized by smooth texture, low TEWL, and high hydration levels [35,36]. In this study, tape stripping resulted in increased TEWL, significantly reduced skin hydration, surface roughness, and epidermal thickening. PFE significantly decreased TEWL, increased skin hydration, and reduced skin roughness, erythema, and epidermal thickness, ultimately leading to a smoother skin appearance.

Targeting skin barrier-related pathways presents considerable complexity due to the multifunctional and interconnected nature of barrier proteins. In this context, proteomics serves as a pivotal tool for elucidating molecular targets of bioactive compounds and therapeutic agents. In this study, we employed proteomic analysis to identify potential molecular targets underlying the efficacy of PFE. The results demonstrated that the barrier-repairing effects of PFE were principally mediated through the modulation of CE and TJs, including Flg2, Lor, and Ivl. These findings provide critical direction for identifying the mechanistic targets through which PFE restores skin barrier function.

The stratum corneum, with its characteristic “brick-and-mortar” organization, constitutes the skin’s primary defensive barrier. In this structure, corneocytes function as the “bricks” that maintain barrier integrity. The CE is the protective outer layer of corneocytes. It is an insoluble and mechanically resilient structure formed through the cross-linking of multiple proteins, including Flg, Lor, and Ivl [37]. Flg is primarily located in the granular and lucid layers of the epidermis, where it degrades into numerous natural moisturizing factors that help retain skin hydration and support barrier integrity. Ivl, situated on the outer layer of the CE, is mainly expressed in the upper spinous and granular layers. As a scaffold protein, it acts as a bridge linking lipids and keratinocytes. Lor, predominantly expressed in the stratum granulosum and stratum corneum, accounts for approximately 60–80% of the total CE protein content [38]. Studies have shown that Lor constitutes the most abundant and resilient CE protein, while Flg is essential for epidermal terminal differentiation and skin barrier formation [39,40]. In summary, Flg, Lor, and Ivl play essential roles in maintaining skin barrier function and homeostasis. This study demonstrates that PFE significantly reverses the downregulation of Flg, Lor, and Ivl at both mRNA and protein levels in a murine model of skin barrier dysfunction, thereby promoting the structural and functional integrity of the CE and ultimately restoring skin barrier function.

TJs are essential components of the skin barrier, interacting with and influencing the expression and function of other barrier structures, such as the microbial and immune barriers of the skin. TJs are cell–cell junctional complexes present in simple, stratified epithelia, and endothelia [20]. They are composed of transmembrane proteins (such as claudin family proteins, tight junction-associated marvel proteins like Ocln, and junctional adhesion molecules (JAMs)), as well as tight junction plaque proteins (such as zonula occludens proteins 1–3, MUPP-1, and cingulin) [41]. TJs primarily seal paracellular pathways, restricting intercellular molecule movement and conferring barrier properties. Additionally, they regulate cell differentiation, proliferation, polarity, and signal transduction [42,43].

The Claudin family constitutes the primary structural component of TJs and acts as a key regulator of paracellular permeability. Decreased claudin expression correlates with elevated TEWL and impaired intercellular adhesion. Studies have shown [44] that newborn mice with claudin-1 knockout exhibit severe dehydration, likely due to excessively high TEWL, and die within 24 h after birth. The Cldn1 downregulation induces skin inflammation and ichthyosis [45]. The occludin protein is predominantly localized to the cell membranes and cytoplasm within the epidermal granular layer. As a transmembrane protein, it plays a critical role in maintaining barrier integrity by anchoring and modulating paracellular permeability. Tjp1 is predominantly localized at the cytoplasmic membrane surface, where it functions as a scaffolding molecule by binding to Cldn and Ocln [46]. TJs play vital roles in intercellular sealing, the compartmentalization of extracellular environments, and preserving the skin barrier’s integrity [47]. Our data demonstrated that PFE significantly alleviated the tape stripping-induced downregulation of Cldn1, Tjp1, and Ocln at both mRNA and protein levels, enhanced intercellular connectivity, reduced transepidermal water loss, and thereby promoted the restoration of skin barrier function.

In summary, this study provides evidence that PFE promotes the recovery of skin barrier integrity through upregulation of CE and TJs after barrier disruption, in parallel with attenuated TEWL and elevated cutaneous hydration. Nevertheless, this work has limitations, as it reveals only a partial set of targets within the associated pathway. Further investigation is necessary to validate additional molecular components and comprehensively elucidate the mechanistic basis of PFE-mediated skin barrier repair.

## 4. Materials and Methods

### 4.1. Chemicals and Reagents

*P.ostii* flowers were supplied by Heze Qishi Co., Ltd. (Heze, Shandong, China). Asiaticoside hydrogel was purchased from Hainan Puli Co., Ltd. (Haikou, Hainan, China). Rhoifolin reference was purchased from the National Institutes for Food and Drug Control (Beijing, China, Cat. No. 111919).

### 4.2. Animals and Treatment Conditions

Beijing Vital River Laboratory Animal Technology Co., Ltd. (Beijing, China) was the source of ICR mice (male, 6–8 weeks, 18–22 g). The Animal Care and Use Committee of China Academy of Chinese Medical Sciences issued approval of the use and care of animals in this work that was in lieu of the Guide for the Care and Use of Laboratory Animals from the National Institutes of Health (No. ERCCACMS21-2406-05). All animals were maintained in a barrier environment under a 12 h light/dark cycle, at a temperature of 23 ± 1 °C, and 50–60% humidity with free access to standard chow (Koao Co., Ltd., Tianjin, China) and water throughout the entire experiment.

### 4.3. Epidermal Barrier Disruption

Dorsal hair was removed from mice using depilatory cream (VEET, Reckitt Benckiser Home Chemicals (China) Co., LTD, Jingzhou, Hubei, China), followed by gentle cleansing with warm water and drying. Tape from 3M (Minnesota Mining and Manufacturing, St. Paul, MN, USA) was applied to the smoothed skin on the mid-dorsum, with its edges marked for reference. Uniform pressure was applied using a D500 press device (compatible with 3M tape) for 10 s per application, repeated approximately twenty times. The modeling area was a circle with a diameter of 2.2 cm. Model validation was assessed via TEWL measurements [19,48].

### 4.4. Preparation of the Hydrogel

An amount of 1.5 g of sodium carboxymethyl cellulose (C8621, Beijing Solaibao Technology Co., LTD, Bejing, China) was accurately weighed, and 43.5 mL of deionized water was slowly added under continuous stirring with a glass rod to prevent clumping. After complete swelling of the polymer, 5 mL of glycerol (G8190, Beijing Solaibao Technology Co., LTD, China) was incorporated, and stirring was continued until a homogeneous mixture was obtained. The gel was then left at room temperature overnight to remove air bubbles and enhance matrix homogenization. PFE solution or deionized water was mixed with the base hydrogel at a 1:1 volume ratio under stirring to obtain uniform PFE hydrogels or blank hydrogel. The asiaticoside hydrogel was commercially available and pre-formulated.

### 4.5. Preparation of PFE

Dried *P. ostii* flowers (100 g) were rinsed with deionized water and transferred to a stainless-steel vessel. Two thousand milliliters of deionized water were added, and the mixture was boiled for 4 h. The extract was filtered through a 500-mesh sieve. The filtrate was refrigerated at 4 °C for 24 h and then subjected to reduced-pressure filtration. The recovered solid was air-dried at ambient temperature (25 ± 2 °C) and pulverized to obtain PFE. The extract was quality-controlled by HPLC (Waters, Milford, MA, USA).

### 4.6. Experimental Design

Mice were randomly divided into six groups: control group (normal mice without any treatment), model group (tape stripping + blank hydrogel), asiaticoside group (tape stripping + 25 mg/mL asiaticoside hydrogel), L-PFE group (tape stripping + 0.06 mg/mL PFE hydrogel), M-PFE group (tape stripping + 0.25 mg/mL PFE hydrogel), and H-PFE group (tape stripping + 1 mg/mL PFE hydrogel). All groups received topical treatments (100 μL per mouse), administered once daily.

On Days 1, 3, and 5 post-treatment, a C-Cube imaging system (PAREX GROUPC, Croix, France) measured erythema index and roughness to evaluate skin barrier damage and repair efficacy. An OCT imaging system (QSLF-1500, Shenzhen MOPTIM Imaging Technique Co., Ltd., Shenzhen, China) assessed epidermal thickness, stratum corneum integrity, and dermo-epidermal junction morphology. TEWL and SH measurements quantified the stratum corneum water diffusion rate and water content, evaluating skin barrier integrity. Twelve hours after the final treatment, all the mice were euthanized. Skin samples were collected and either frozen in liquid nitrogen or fixed in 4% or 10% buffered paraformaldehyde for subsequent analysis.

### 4.7. Histological Analysis

For histological analysis, dorsal skin samples were fixed overnight in 4% paraformaldehyde, embedded in paraffin, and sectioned at a thickness of 10 μm. Tissue sections were stained with hematoxylin and eosin (H&E) and examined microscopically (CKX41, Olympus Corporation, Tokyo, Japan). H&E staining enabled visualization of epidermal and dermal layer thickness, along with cellular morphology and size.

### 4.8. Assessment of Skin Surface Characteristics

TEWL and SH were analyzed using a Vapometer^®^ SWL5911 (Delfin Technologies, Kuopio, Finland) and a MoistureMeter^®^ MSC1350 (Delfin Technologies, Kuopio, Finland), respectively. Measurements were conducted under controlled environmental conditions (21–22 °C; 50–55% relative humidity). Data are expressed as mean ± SD in units of g·m^−2^·h^−1^ (TEWL) and arbitrary units (AU) (SH). Skin barrier impairment is indicated by a significant increase in TEWL values and a concurrent decrease in SH values.

### 4.9. Immunofluorescence Analysis

Dorsal skin samples were fixed in 10% neutral buffered formalin, embedded in paraffin, and sectioned at a thickness of 6 µm. For immunofluorescence, sections were incubated in an immune staining permeation solution (Boster Biological Technology Co., Ltd., Wuhan, China) for 5 min and then blocked with immunofluorescence blocking solution (Boster Biological Technology Co., Ltd., Wuhan, China) for 10 min. After that, the sections were incubated overnight at 4 °C with the following primary antibodies diluted in antibody diluent: anti-Flg (Santacruz, Dallas, TX, USA, J2221, 1:150), anti-Ivl (Abclonal, Woburn, MA, USA, A13311, 1:80), anti-Lor (Abclonal, A21039, 1:80), anti-Cldn1 (Abclonal, A11530, 1:80), anti-Tjp1 (Abclonal, A25306, 1:80), and anti-Ocln (Abclonal, A25320, 1:80). After three washes with TBS, the sections were incubated with species-appropriate fluorescent secondary antibodies according to the isotype of the primary antibodies. The secondary antibodies were diluted as follows: anti-rabbit (Boster, Pleasanton, CA, USA, BA1105, 1:80) and anti-mouse (Boster, BA1101, 1:80), both in antibody diluent, and incubated at room temperature for 1 h. The sections were then washed three times with 1× TBS for 5 min each at room temperature. Nuclei were counterstained with 4′,6-diamidino-2-phenylindole (DAPI). Finally, all stained sections were examined using an FV1000MPE multiphoton microscope (Olympus Corporation, Tokyo, Japan) to assess marker localization and histological changes.

### 4.10. qRT-PCR

Total RNA was extracted from samples using Trizol reagent (TOYOBO Biotechnology Co., Ltd., Shanghai, China) according to the manufacturer’s protocol. cDNA synthesis was performed from 1 μg total RNA using the FastKing cDNA Dispelling RT SuperMix kit (Tiangen Biotech Co., Ltd., Beijing, China). A Bio-Rad CFX96 system (Bio-Rad, Hercules, CA, USA) was utilized for qRT-PCR employing the SYBR Green Master Mix (Applied Biosystems, Foster City, CA, USA). Primer sequences are listed in Table 1.

Relative mRNA expression was calculated using the 2^−ΔΔCt^ method, with Gapdh as the endogenous control. All reactions were performed in triplicate.

### 4.11. Proteome Analysis

The sample preparation process involved protein denaturation, reduction, alkylation, tryptic digestion, and peptide clearance. First, samples were homogenized in 200 μL UA buffer (8 M urea, 0.1 M Tris/HCl, pH 8.5). The pH was adjusted by adding 100 mM NH_4_HCO_3_. Reduction was performed by adding 0.25 μL of 1 M dithiothreitol followed by incubation at 37 °C for 4 h. Alkylation was then carried out by adding 1.25 μL of 1 M iodoacetamide and incubating in the dark at room temperature for 1 h. Any excess iodoacetamide was quenched by adding 0.5 μL of 1 M dithiothreitol. Add 0.5 μL of 1 M dithiothreitol to quench the remaining iodoacetamide. Subsequently, trypsin (Promega, Madison, WI, USA) in digestion buffer was added. Samples were incubated at 37 °C with shaking (500 rpm) for 2 h, followed by overnight incubation at 37 °C. Formic acid (FA) was added to a final concentration of 1%. The sample was centrifuged at 12,000× *g* for 5 min, and the supernatant was then centrifuged in SEP-PAK VAC C18 cartridges (Waters, Milford, MA, USA), which were pre-activated by 0.1% FA. This process resulted in the absorption of peptides obtained after enzymolysis onto the C18 cartridges. The C18 cartridges were washed with 50% acetonitrile and 0.1% TFA, and the desorbed purified peptide solution was obtained. The purified peptide segments were freeze-dried and stored at −20 °C. Before analysis, the samples were reconstituted with an injection buffer and subjected to DIA data acquisition using an Acquity M-class HPLC system (Waters, Milford, MA, USA) coupled to a ZenoTOF 7600 mass spectrometer (Sciex, Redwood City, CA, USA) equipped with an OptiFlow Turbo V ion source and powered by SciexOS 3.1 software (AB Sciex version 2015). Finally, the DIA-NN software (version 1.8.1) was used for data analysis to obtain protein qualitative and quantitative results.

### 4.12. Statistical Analysis

The results are presented as the mean ± SD, and calculations were performed using GraphPad Prism 7.0 (GraphPad Software, San Diego, CA, USA). The one-way ANOVA with Tukey’s analysis was used to determine statistical significance between different groups, and *p* < 0.05 was considered statistically significant.

## 5. Conclusions

Our study integrates proteomic analysis with experimental validation to elucidate, for the first time, the mechanism underlying PFE-mediated skin barrier repair. These findings provide valuable insights to guide the future development of PFE as a therapeutic topical cosmetic for combating skin barrier impairment.

## Figures and Tables

**Table 1:** Primer sequences.

Gene	Primer Direction	Sequence (5′→3′)
*Flg*	Forward	GCAAGATCAGGCTCAGGAGGAAG
	Reverse	AGAATGGACTTGGCTGTCACTGG
*Lor*	Forward	GGCGGCGGCGGCTATTATAG
	Reverse	GGAACCACCTCCATAGGAACCAC
*Ivl*	Forward	GGGACCTCTCAAGACTGTGTGTTC
	Reverse	AGACCTGGCATTGTGTAGGATGTG
*Tjp1*	Forward	ACCCGAAACTGATGCTGTGGATAG
	Reverse	GCTGGCTGGCTGTACTGTGAG
*Cldn1*	Forward	GTGTCCTACTTTCCTGCTCCTGTC
	Reverse	AGAAGGTGTTGGCTTGGGATAAGG
*Ocln*	Forward	CCTCTGACCTTGAGTGTGGATGAC
	Reverse	TCCTCTTGCCCTTTCCTGCTTTC
*Gapdh*	Forward	AGAAGGTGGTGAAGCAGGCATC
	Reverse	CGAAGGTGGAAGAGTGGGAGTTG

## Data Availability

The manuscript did not generate new publicly archived data. All relevant data of the current study can be requested from the authors.

## Abbreviations

TEWL	transepidermal water loss
PFE	peony flower extract
IF	immunofluorescence
CE	cornified envelope
TJs	tight junctions
FASP	filter-aided sample preparation
PPI	protein-protein interaction
TCM	traditional Chinese medicine
Flg	filaggrin
Ivl	involucrin
Lor	loricrin
Cldn1	claudin-1
Tjp1	zonula occludens-1
Ocln	occludin
DAPI	4′,6-diamidino-2-phenylindole
FA	Formic acid
HPLC	high-performance liquid chromatography
